# *Helicobacter suis* affects the health and function of porcine gastric parietal cells

**DOI:** 10.1186/s13567-016-0386-1

**Published:** 2016-10-19

**Authors:** Guangzhi Zhang, Richard Ducatelle, Belgacem Mihi, Annemieke Smet, Bram Flahou, Freddy Haesebrouck

**Affiliations:** 1 Department of Pathology, Bacteriology and Avian Diseases, Faculty of Veterinary Medicine, Ghent University, Merelbeke, Belgium; 2 Department of Virology, Parasitology and Immunology, Faculty of Veterinary Medicine, Ghent University, Merelbeke, Belgium; 3 Department of Immunology, Graduate School of Medicine, Chiba University, Chiba, Japan; 4 Department of Molecular and Cell Biology, University of California, Berkeley, USA

## Abstract

The stomach of pigs at slaughter age is often colonized by *Helicobacter* (*H.*) *suis*, which is also the most prevalent gastric non-*H. pylori Helicobacter* (NHPH) species in humans. It is associated with chronic gastritis, gastric ulceration and other gastric pathological changes in both hosts. Parietal cells are highly specialized, terminally differentiated epithelial cells responsible for gastric acid secretion and regulation. Dysfunction of these cells is closely associated with gastric pathology and disease. Here we describe a method for isolation and culture of viable and responsive parietal cells from slaughterhouse pigs. In addition, we investigated the interactions between *H. suis* and gastric parietal cells both in *H. suis*-infected six-month-old slaughter pigs, as well as in our in vitro parietal cell model. A close interaction of *H. suis* and parietal cells was observed in the fundic region of stomachs from *H. suis* positive pigs. The bacterium was shown to be able to directly interfere with cultured porcine parietal cells, causing a significant impairment of cell viability. Transcriptional levels of Atp4a, essential for gastric acid secretion, showed a trend towards an up-regulation in *H. suis* positive pigs compared to *H. suis*-negative pigs. In addition, sonic hedgehog, an important factor involved in gastric epithelial differentiation, gastric mucosal repair, and stomach homeostasis, was also significantly up-regulated in *H. suis* positive pigs. In conclusion, this study describes a successful approach for the isolation and culture of porcine gastric parietal cells. The results indicate that *H. suis* affects the viability and function of this cell type.

## Introduction

*Helicobacter* (*H*.) *suis* is a Gram-negative bacterium with a typical spiral-shaped morphology, which frequently colonizes the stomach of pigs as well as a minority of humans [1–3]. Indeed, gastric non-*H. pylori* helicobacters (NHPH) are found in 0.2–6% of gastric biopsies, depending on the study [4], and *H. suis* is considered to be the most prevalent NHPH in humans [3–5]. In humans, infection with *H. suis* has been described to cause gastritis, gastric ulceration, as well as gastric mucosa-associated lymphoid tissue (MALT) lymphoma and sporadically gastric adenocarcinoma [6–8]. In naturally infected or experimentally infected pigs, *H. suis* infection has been shown to cause gastritis, reduced daily weight gain and other gastric pathological changes [9, 10].

The gastric mucosa is composed of various cell types. Parietal (oxyntic) cells are abundant in the fundic gland region. They are responsible for the secretion of gastric acid and play a vital role in the maintenance of the normal structure and function of the gastric mucosa [11]. In some species, including humans, pigs, rabbits and cats, parietal cells can also secrete intrinsic factor which plays an important role in the absorption of vitamins and other nutrients by the small intestine [12]. Hydrogen potassium ATPase (H^+^/K^+^ ATPase) is the proton pump composed of a catalytic subunit (α-subunit) and an accessory subunit (β-subunit) in parietal cells, and it mediates secretion of acid into the gastric lumen [11]. Various studies have shown that atrophic gastritis induced by *H. pylori* infection is characterized by the dysfunction or loss of parietal cells [13, 14]. While *H. pylori* is mainly observed in the mucus layer or close to mucus-producing cells, *H. suis* is often observed near or even inside the canaliculi of parietal cells in experimentally infected Mongolian gerbils and mice. Similar observations have been made in humans [15]. Both in rodent models and humans, these parietal cells can show signs of degeneration [15, 16].

Besides H^+^/K^+^ ATPase, sonic hedgehog (Shh) is another identified factor playing an important role in the regulation of gastric acid secretion, as well as maturation and differentiation of gastric epithelial cells and fundic glands in mice and humans under normal conditions [17, 18]. It has also been described to play a role in the pathogenesis of *H. pylori* infection and in the development of gastric cancer [19, 20]. Currently, no information is available on potential effects of *H. suis* infection on the expression of Shh.

To date, there is no report illustrating the interactions between *H. suis* and parietal cells in pigs. Therefore, the aim of this study was to examine the direct effects of *H. suis* on porcine parietal cells, both using a newly developed in vitro parietal cell culture method and tissues from *H. suis*-infected pigs.

## Materials and methods

### Collection of pig stomachs

All pig stomachs were collected from 6 month-old slaughter pigs, brought to the laboratory immediately, and kept at 4 °C until further use.

### Isolation and culture of primary porcine parietal cells

Pig stomachs were opened, and washed successively several times with water (37 °C) and phosphate buffered saline (PBS; 37 °C). The mucus was removed using a glass slide, and the fundic region of the stomach was collected and kept in ice-cold PBS. The mucosa was separated gently from the underlying tunica submucosa and tunica muscularis, using the sharp side of a scalpel, and minced into small fragments. After washing the minced mucosa several times with PBS (37 °C) and minimal essential medium-glutamax (37 °C) (MEM; Invitrogen, Carlsbad, CA, USA), it was placed in MEM supplemented with dispase (1 mg/mL, Invitrogen) and BSA (5 mg/mL). This mixture was transferred to a tissue culture flask, and the tissue was digested at 37 °C for 25 min on a rotational shaker. The digestion was stopped by three-fold dilution with MEM, and the sample was subjected to centrifugation at 200 *g* for 10 min. The supernatant was discarded and the tissue was placed in MEM supplemented with collagenase type 1 (2.5 mg/mL, Invitrogen) and BSA (5 mg/mL) and incubated for another 50 min under the same conditions as described above. The resulting mixture was filtered through a 150 µm metal sieve, and centrifuged at 200*g* for 10 min. The supernatant was removed carefully. The remaining cells were washed with MEM, and then filtered using a 70 and 40 µm cell strainer for two times each. The cell suspension was washed two times in MEM, and further purified using an OptiPrep™ gradient (Sigma-Aldrich St. Louis, MO, USA) according to the procedure described by Chew and Brown [21]. The purified cells were washed in MEM and incubated in cell culture flasks containing medium A [DMEM/F12 (Sigma-Aldrich) supplemented with 20 mM Hepes, 0.2% BSA, 10 mM glucose, 8 nM EGF (Sigma-Aldrich), 1× Insulin, Transferrin, Selenium Solution (ITS) (Invitrogen), 1% penicillin–streptomycin, 50 μg/mL amphotericin B and 25 μg/mL gentamicin (Invitrogen)] for 40 min to eliminate contaminating bacteria and fungi. Subsequently, the cells were washed in DMEM/F12 supplemented with 0.2% BSA and 10 mM glucose, and incubated in medium A without amphotericin B in 24-well flat-bottom cell-culture plates (Greiner Bio-One, Frickenhausen, Germany) containing Matrigel^®^-coated glass coverslips (circular diameter 12 mm; Thermo Scientific, Leicestershire, UK). To coat these coverslips, Matrigel^®^ basement membrane matrix (Corning B.V. Life Sciences, Amsterdam, LJ, Netherlands) was thawed on ice for at least 12 h. Subsequently, the glass coverslips were coated with Matrigel^®^ matrix, diluted six times in ice-cold sterile water, and left to dry in a laminar air flow over night.

### Activation of parietal cells and visualization of gastric acid secretion

Twelve hours after seeding of parietal cells on coverslips, the medium was replaced by fresh medium. In order to stimulate cells to secrete HCl, they were incubated in medium supplemented with histamine (400 μM; Sigma-Aldrich) and 3-isobutyl-1-methylxanthine (IBMX) (30 μM; Sigma). Control cells were held in a resting state by administering cimetidine (100 μM; Sigma-Aldrich). After 30 min of incubation at 37 °C, cells were incubated in medium A without amphotericin B and supplemented with 2 µM LysoSensor™ Yellow/Blue DND-160 (Invitrogen) and 2 µM Cell Tracker Red CMTPX (Invitrogen) at 37 °C for 30 min. Subsequently, cells were washed 3 times, immediately mounted in a small volume of PBS (50% glycerol, v/v) on glass slides at room temperature, and analyzed using a confocal microscopy within 30 min.

### Preparation of *H. suis* and bacterial lysate

*H. suis* strain HS5cLP was grown on Brucella agar (BD, Franklin Lakes, NJ, USA) plates with a pH of 5 and supplemented with 20% fetal calf serum (HyClone), 5 mg/L amphotericin B (Fungizone; Bristol-Myers Squibb, Epernon, France), Campylobacter selective supplement (Oxoid, Basingstoke, UK) and Vitox supplement (Oxoid) under microaerobic and biphasic conditions (37 °C; 85% N_2_, 10% CO_2_, 5% O_2_) as described elsewhere [22]. This strain was isolated in 2008 from the stomach of a slaughterhouse pig [23]. Bacterial lysate was prepared as described previously [24].

### Treatment of parietal cells and determination of cell viability

Parietal cells were cultured as described above in fresh medium without antibiotics and amphotericin B. Parietal cells were inoculated with viable *H. suis* bacteria at a multiplicity of infection (MOI) of 100 or 200 or with whole bacterial lysate at a final concentration of 100 µg/mL or 200 µg/mL in 24-well plates. Parietal cells incubated with Hank’s buffered salt solution (HBSS) with Ca^2+^ and Mg^2+^ (Gibco, Life Technologies, Paisley, Scotland) were used as controls. For the first 4 h, incubation was done at 37 °C under microaerobic conditions, after which the cells were transferred to normal conditions (5% CO_2_) for another 20 h. Parietal cell viability was determined using the neutral red (3-amino-7-dimethylamino-2-methyl-phenazine hydrochloride) uptake assay as described previously with some minor modifications [25]. Briefly, 400 μL of pre-warmed neutral red solution (33 μg/mL in DMEM without phenol red) was added to each well and the plate was incubated at 37 °C for 3 h. The cells were then washed twice with HBSS. Two hundred microliter of extracting solution [ethanol/water/acetic acid, 50/49/1 (v/v/v)] was added to each well to release the dye, and the plate was shaken for another 30 min. The absorbance was then read at 540 nm with a microplate ELISA reader (Multiscan MS, Thermo Labsystems, Helsinki, Finland). The percentage of viable cells was estimated using the following formula:

$\% {\text{ cell viability}} = 100\; \times ({\text{a}} - {\text{b}})/({\text{c}} - {\text{b}})$ with a = OD_540_ derived from the wells incubated with live bacteria or lysate, b = OD_540_ derived from blank wells, c = OD_540_ derived from untreated control wells.

### Indirect immunofluorescent staining

Cultured parietal cells treated as described above were fixed with 4% paraformaldehyde in PBS for 15 min at room temperature. After fixation, the cells were washed three times with PBS, and permeabilized with 0.3% Triton X-100 in PBS (2% BSA) for 20 min followed by incubation in PBS (2% BSA) for another 30 min. The cells were washed 3 times with PBS. Subsequently, cells were incubated with a primary mouse monoclonal anti-H^+^/K^+^ ATPase β-subunit antibody (1/200; Abcam Ltd, Cambridge, UK) and a polyclonal rabbit anti-*H. pylori* antibody (1/320; Dako, Glostrup, Denmark) for 1 h at 37 °C, followed by an Alexa Fluor 633-conjugated goat anti-mouse secondary antibody (1/200; Invitrogen) and Alexa Fluor 488-conjugated goat anti-rabbit IgG (1/100; Invitrogen) for 1 h at 37 °C. All antibodies were diluted in PBS and the cells were washed 5 times after incubation with the primary and secondary antibodies. Incubation for 15 min with DAPI (0.5 μg/mL; Sigma) was performed to counterstain the nuclei and the cells were rinsed 5 times in PBS. Stained cells were mounted in ProLong ^®^ Gold antifade reagent medium (Invitrogen) and imaged by an Olympus BX61 fluorescence microscope (Olympus Belgium N.V.).

### Immunohistochemical (IHC) and immunofluorescent staining of pig gastric tissue slides

Stomachs from slaughterhouse pigs were opened along the greater curvature. For detection of *H. suis* colonization, a small piece of tissue from the fundic region of the stomach was collected, followed by DNA extraction and *H. suis*-specific Quantitative Real-Time PCR (qRT-PCR) as described previously [26].

Gastric samples from the fundic gland zone were fixed in 10% phosphate-buffered formalin, processed by routine methods and embedded in paraffin. Consecutive sections of 5 µm were cut, and IHC staining for the identification and visualization of parietal cells was performed with these sections as described previously [16]. Immunofluorescent staining was also performed to visualize co-localization of parietal cells and *H. suis*. Briefly, 5 μm formaldehyde-fixed tissue sections were deparaffinized in xylene and rehydrated in graded ethanol. Sections were boiled in antigen retrieval solution (850 W, 1.5 min; 300 W, 10 min) and washed respectively for 15 min in water and 5 min in PBS. Sections were permeabilized with 0.3% TritonX-100 in PBS (2% goat serum) for 15 min, and incubated in PBS (10% goat serum) for 45 min. Tissue sections were incubated with a primary mouse monoclonal anti-H^+^/K^+^ ATPase β-subunit antibody (1/3125; Abcam) and a polyclonal rabbit anti-*H. pylori* (1/320; Dako) antibody overnight at 4 °C. After washing with PBS, sections were incubated for 1 h with secondary Alexa Fluor 633 goat anti-mouse IgG (1/100; Invitrogen) and Alexa Fluor 488 goat anti-rabbit IgG (1/100; Invitrogen). DAPI (0.5 μg/mL) was used to counterstain the nuclei. Tissue sections were washed extensively with PBS, mounted in ProLong^®^ Gold antifade reagent medium and examined by fluorescence or confocal microscopy.

### RNA extraction, reverse transcription and qRT-PCR

qRT-PCR was used to compare gene expression levels of gastric tissue from *H. suis* negative pigs (*n* = 15) and *H. suis* positive pigs (*n* = 15). RNA was extracted and cDNA was prepared as described previously [22]. Total RNA was extracted using the RNeasy Mini Kit (Qiagen, Hilden, Germany) according to the manufacturer’s instructions. The concentration of RNA was measured using a NanoDrop spectrophotometer (Isogen Life Science, PW De Meern, Utrecht, The Netherlands). The purity of the RNA was evaluated with the Experion automated electrophoresis system using StdSens RNA chips (Bio-Rad, Hercules CA, USA). The RNA concentration from all samples was adjusted to 1 μg/μL and cDNA was synthesized immediately using the iScript™ cDNA Synthesis Kit (Bio-Rad).

The housekeeping genes *ACTB, Cyc*-5 and *HPRT* were included as reference genes (Bosschem et al. unpublished data). Primers for *Atp4a* were referenced elsewhere [27], and primers for *Shh* were designed based on the conserved complete or partial coding sequences of *Shh* available for humans, pigs, mice and rats on GenBank. The mRNA expression levels of reference genes and target genes were quantified using SYBR Green based RT-PCR with iQ™ SYBR Green Supermix. Reactions were performed using a CFX96 RT PCR System in a C1000 Thermal Cycler (Bio-Rad). qRT-PCR was performed as described elsewhere [22]. Sequence information of the primers is shown in Table 1.

**Table 1:** **Primers used in qRT-PCR**

Gene	Primer	Sequence (5′-3′)	Reference
*Atp4a*	Sense	GCATATGAGAAGGCCGAGAG	[27]
	Antisense	TGGCCGTGAAGTAGTCAGTG	
*Sonic hedgehog*	Sense	TGACCCCTTTAGCCTACAAGCA	This study
	Antisense	TGGGGGTGAGTTCCTTAAATCG	

## Results

### Activation of parietal cells and stimulation of gastric acid secretion

Cultured parietal cells responded to stimulation with histamine/IBMX, as shown by the presence of more and bigger vacuoles observed by light microscopy (data not shown). In order to confirm the secretion of gastric acid by parietal cells after stimulation, a fluorescent acidic pH indicator, LysoSensor, was loaded both to resting and stimulated parietal cells.

**Figure 1:**
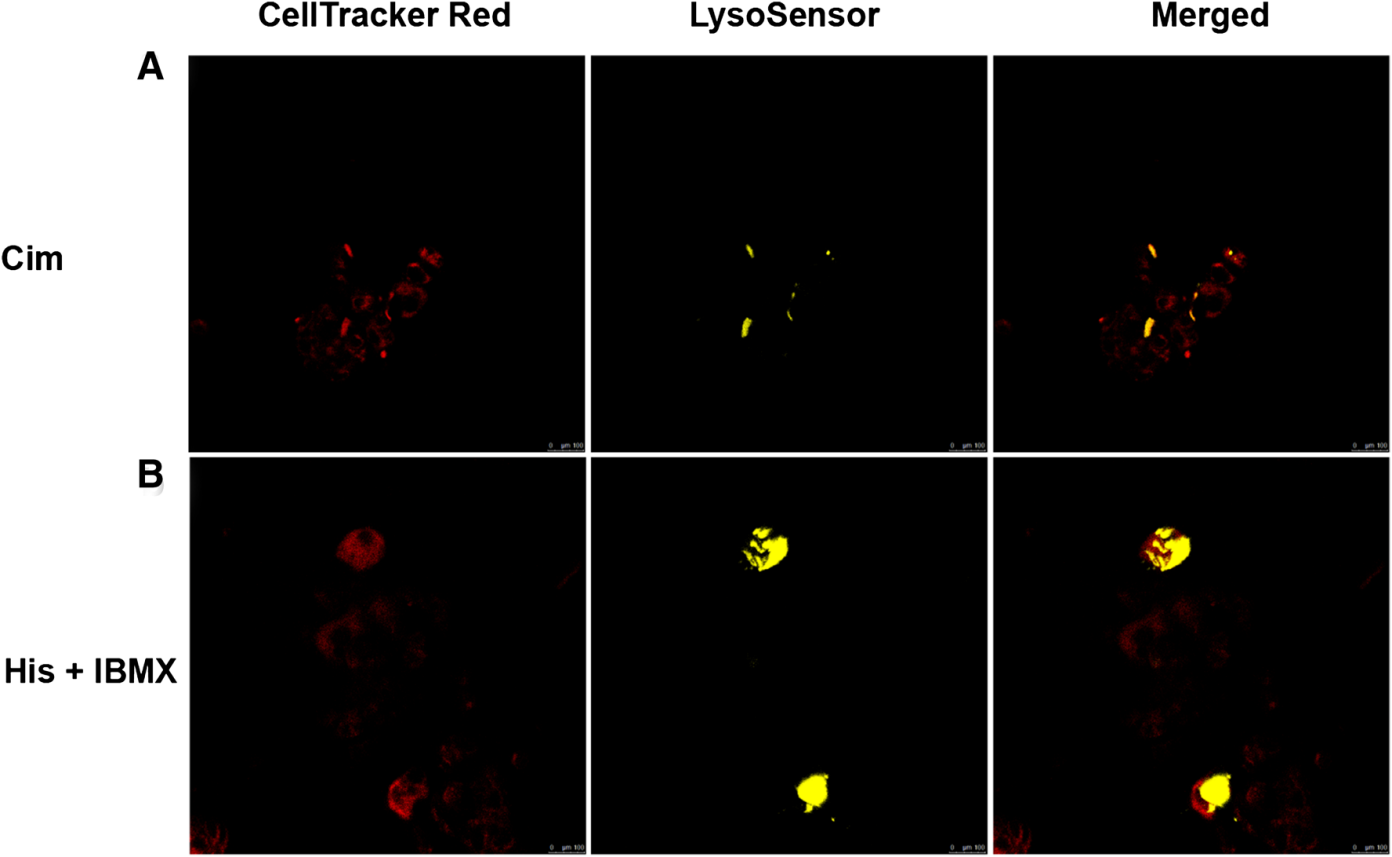
**Acid secretion by parietal cells.** LysoSensor™ Yellow/Blue DND-160 was used to monitor the acid secretion by live parietal cells incubated either by Cim or His/IBMX. Cell Tracker Red CMTPX was used to track all live cells. Some apical vacuoles of parietal cells in resting stage revealed several small areas with weak yellow fluorescence, indicating basal acid secretion (**A**). Parietal cell treated with His/IBMX showed an expansion of apical vacuoles, as shown by strongly enlarged zones with yellow fluorescence, indicating the continuous secretion of acid (**B**). Cim: Cimitidine; His: histamine; IBMX: 3-isobutyl-1-methylxanthine.

An accumulation of LysoSensor was observed in the stimulated parietal cells, characterized by a strong yellow fluorescence (Figure 1B). Parietal cells in resting stage also showed several small areas with weak yellow fluorescence, indicating that vacuoles in parietal cells had a basal acid production (Figure 1A). Upon the stimulation by histamine*/*IBMX, an increase in the fluorescence intensity of LysoSensor in the vacuoles was observed and the size of the vacuoles was increased as well (Figure 1B), indicating the enhancement of gastric acid secretion.

### *H. suis* bacteria interact with cultured parietal cells

**Figure 2:**
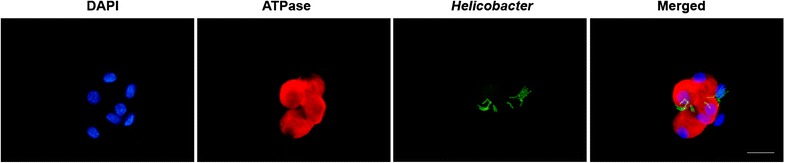
**The presence of** ***H. suis*** **near or inside the cultured parietal cell.** Cultured parietal cells were inoculated with live *H. suis* at an MOI of 10 for 6 h, and a close relationship between *H. suis* (green) and parietal cells (red) could be observed. Cell nuclei were stained by DAPI (blue). Representative fluorescence micrographs were shown. Scale bars: 50 µm. MOI: multiplicity of infection.

Immunofluorescence staining showed adhesion of *H. suis* to parietal cells after incubation of cells with *H. suis* at an MOI of 10 or 100:1 for 6 h (Figure 2). Longer incubation time (12 h) and a higher MOI (200) exhibited similar results (data not shown).

### Cell viability assay

**Figure 3:**
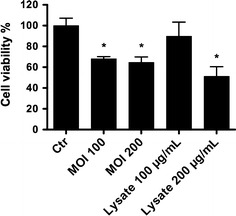
**Effect of** ***H. suis*** **on parietal cell viability.** Parietal cells were treated with live *H. suis* (MOI: 100:1, 200:1) or whole bacterial lysate (100 µg/mL, 200 µg/mL), and control cells were treated with HBSS. After 24 h, cell viability was determined by a neutral red assay. Results of one representative experiment (out of 3 performed in total) are shown (*n* = 5). An * represents a statistically significant difference between bacteria or lysate treated cells and HBSS treated cells (Student *t* test, *p* < 0.05). MOI: multiplicity of infection; HBSS: Hank’s buffered salt solution.

A neutral red assay was used to determine the effect of live *H. suis* bacteria and whole cell lysate of *H. suis* on parietal cell viability. Parietal cells were treated with live bacteria or bacterial lysate for 24 h. Compared to untreated control cells, a significant decrease of cell viability was observed in live bacteria-treated cells (MOI: 100, 200) and lysate-treated cells (200 µg/mL) (Figure 3, *p* < 0.05), confirming that both live bacteria and lysate can affect parietal cell viability in vitro.

### Interaction between *H. suis* and porcine parietal cells in vivo

**Figure 4:**
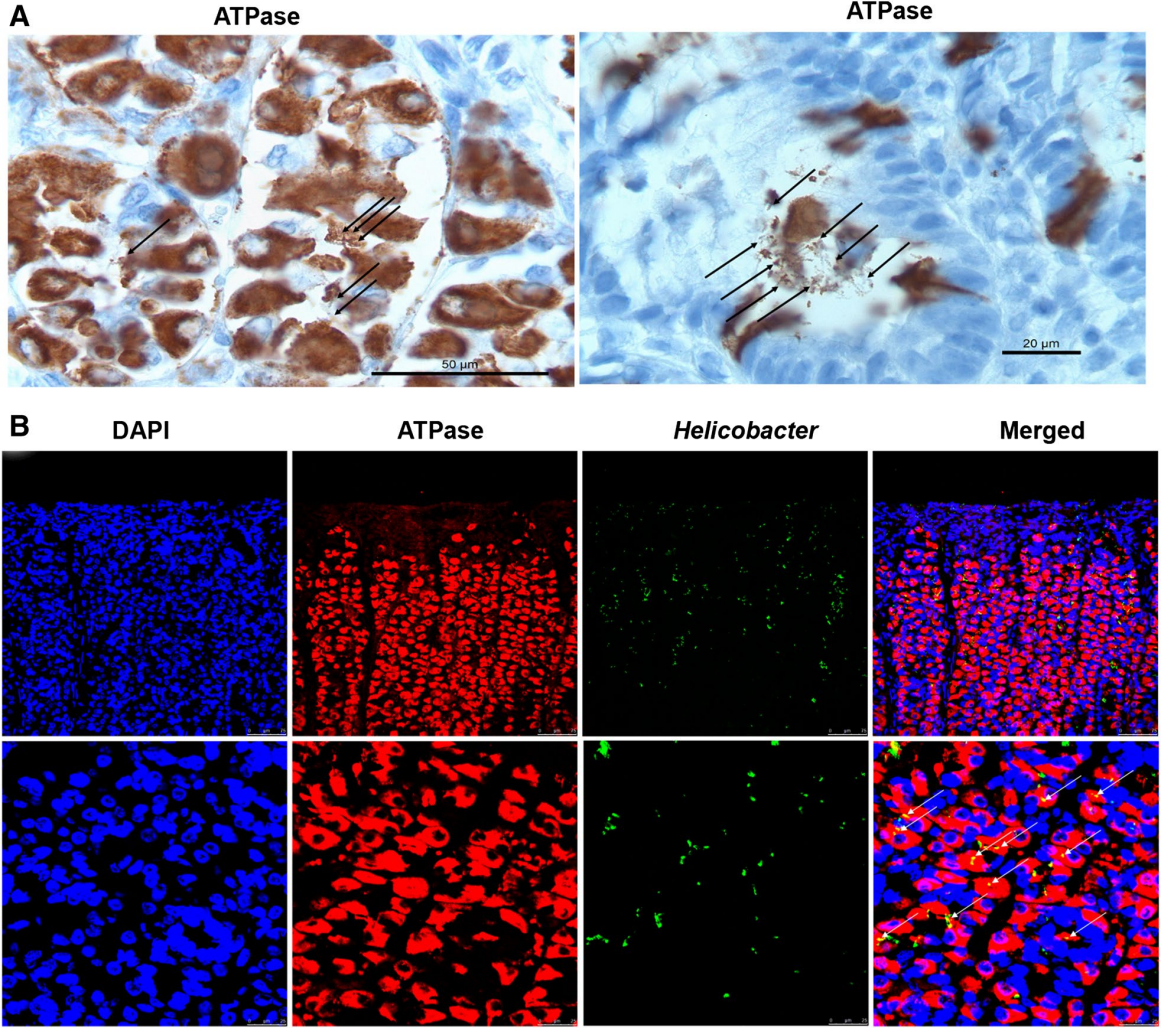
**Co-localization of** ***H. suis*** **and porcine parietal cells in the stomach from slaughterhouse pigs.** Shown are representative IHC (**A**) and fluorescent micrographs (**B**) of *H. suis* bacteria near or potentially in the canaliculi of the parietal cells in the fundic region of the stomach from slaughterhouse pigs. IHC staining (**A**) showed that *H. suis* bacteria (black arrows) were observed next to parietal cells (brown), sometimes showing signs of degeneration (right panel). Immunofluorescence staining (**B**) revealed that a substantial number of *H. suis* bacteria (green, white arrows) can be found in close association or in the canaliculi of the parietal cells (red). Nuclei are stained with DAPI (blue). IHC: immunohistochemistry.

IHC staining did not reveal a clear change of parietal cell numbers in the stomach of *H. suis*-infected pigs compared to *H. suis*-negative pigs (data not shown). However, a close relationship between parietal cells and *H. suis* was observed in the fundic region of the pig stomach (Figure 4A), and *H. suis* bacteria could also be observed amidst the debris of parietal cells (Figure 4A, right panel). In order to further investigate the co-localization of parietal cells and *H. suis*, a double immunofluorescence staining for H^+^/K^+^ ATPase and *H. suis* was performed. Confocal microscopic analysis showed that the majority of the bacteria were observed in the vicinity of or inside the canaliculi or cytoplasm of parietal cells (Figure 4B).

### qRT-PCR

**Figure 5:**
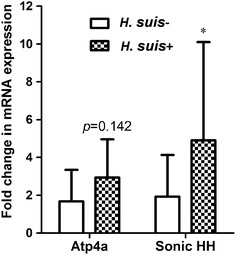
**mRNA expression analysis.** Shown are the mean fold changes of mRNA expression in *H. suis* positive pigs (*n* = 15) for *Atp4a* and *Sonic HH*, compared to that in *H. suis* negative pigs (*n* = 15). An * indicates a statistically significant difference (Student *t* test, *p* < 0.05) between *H. suis* positive pigs (*H. suis*−) and *H. suis* negative pigs (*H. suis*+). Sonic HH: sonic hedgehog.

Transcriptional changes of crucial genes involved in parietal cell function and gastric epithelial cell homeostasis were determined using qRT-PCR. A tendency towards an up-regulation of *Atp4a* was observed in *H. suis* positive pigs compared to negative animals (Figure 5, *p* = 0.14). Compared to *H. suis* negative pigs, a significant up-regulation of *Shh* was observed in *H. suis*-infected slaughter pigs (Figure 5, *p* = 0.012).

## Discussion

Pig stomachs are frequently inhabited by *H. suis*, a zoonotic bacterium, raising concerns regarding animal welfare, economic interests, public health and food safety [4, 10, 28]. *H. suis* infection can cause a decreased body weight gain and gastritis in pigs [10], and chronic gastritis, peptic ulceration and the development of MALT lymphoma-like lesions in humans and rodent models of human gastric disease [8, 16, 29, 30]. In the latter, a close association between *H. suis* and parietal cells has been observed and these cells can show signs of degeneration or malfunction [16]. In previous studies, it has been described that malfunction of acid secretion by parietal cells is closely associated with the development of gastritis [31], indicating that the function of parietal cells might be influenced by gastritis. On the other hand, a direct effect of *H. suis* on the health and function of parietal cells might also be involved. At the onset of this study, very little information was available on the interactions between *H. suis* and parietal cells in its natural host, the pig.

In the present study, we explored and described an effective method for isolation and culture of porcine parietal cells. This cell type is highly specialized and differentiated, requiring a specific approach. Our method was based on previously described methods for the isolation of rabbit parietal cells [32], and to a lesser extent on those described for dogs, rats and mice [33–35]. At first, we followed the protocols described for isolation of rabbit parietal cells, however without a great deal of success. Compared to rabbit stomach mucosa, it is more difficult to separate the pig stomach mucosa from the deeper layers, enzymatic digestion is less efficient, and the mucosa is covered by a thick layer of mucus, all of which give rise to some obstacles during the initial isolation of parietal cells. Some reagents that have previously been shown to be useful for the removal of mucus, including N-acetylcysteine and DTT [36, 37], did not contribute a lot to successful parietal cell isolation in the current study. In addition, some studies have shown that the use of EDTA can disrupt tight junctions between gastric epithelial cells, further facilitating the release of parietal cells from the gastric glands. In our study, however, the administration of EDTA did not exhibit beneficial effects. In view of the existing difficulties, we have optimized some steps that appeared to be essential for isolation of porcine parietal cells. These include an adequate removal of mucus by scraping, separating the mucosa in small pieces from the underlying tissue using a sharp blade and taking care to minimize the presence of submucosa and other connective tissues. Finally, using a combination of dispase and collagenase also proved to contribute to the release of parietal cells from the mucosa. Several matrices were tested for their ability to stimulate adhesion of parietal cells to coverslips, including fibronectin, collagen type I, collagen type IV, gelatine and Matrigel (data not shown). The latter was shown to provide the best results. In general, the majority of the cultured parietal cells existed in the form of single cells or small cellular clumps, and they were shown to remain viable under the described conditions for up to 5 days with a purity of ~80%.

In the present study, histological analysis of the stomachs of *H. suis*-infected pigs at slaughter age, revealed that *H. suis* bacteria are often observed in close vicinity of parietal cells and they even can be observed inside the canaliculi of parietal cells, which reveals a direct interaction of *H. suis* and parietal cells in situ. Upon co-incubation of isolated parietal cells with live *H. suis*, a considerable number of *H. suis* bacteria were found near or potentially in the canaliculi of the isolated parietal cells, which further confirmed the direct interplay between this bacterium and parietal cells in vivo and in vitro. Longer times of incubation of *H. suis* with isolated parietal cells showed similar results, and the most plausible explanation for this may be that a longer incubation time decreases the bacterial viability due to the improper medium and gas environment for this fastidious bacterium, requiring vigorous culture conditions. Future experiments should attempt to identify the possible mechanisms of adhesion.

IHC and immunofluorescent analysis revealed that *H. suis* infection did not greatly affect parietal cell numbers in the stomach of naturally infected pigs. We were, nevertheless, able to show for the first time a direct effect of *H. suis* on the viability of cultured parietal cells. This confirms previous findings that long-term *H. suis* infection can induce necrosis of parietal cells in the stomach of experimentally infected mice and Mongolian gerbils [16] and that swollen and degenerated parietal cells are often found in NHPH-infected patients with chronic gastritis [15]. Future experiments should aim to characterize the mechanisms involved. For *H. pylori*, it has been shown that infection can induce apoptosis of cultured rat parietal cells in a nuclear factor-κB- and nitric oxide-dependent manner [38].

Other gastric *Helicobacter* species, including *H. pylori*, *H. heilmannii*, and *H. felis*, have been described to cause massive parietal cell loss in rodent models, leading to the deregulation of gastric morphology and the development of intestinal metaplasia [39–41]. Most likely, the development of gastritis in the corpus region, which is more pronounced compared to *H. suis* infection in these same animal models, contributes largely to this massive loss of parietal cells. Indeed, Feldman et al., have demonstrated a positive correlation between the severity of *H. pylori*-related corpus gastritis and the degree of reduction in acid secretion function of parietal cells [42], and other reports have shown that the development of chronic gastritis in patients with *H. pylori* infection is associated with or causes the loss of parietal cells [43–45].

Besides an effect on the viability of parietal cells, *H. suis* may also affect the normal function and homeostasis of parietal cells in particular and the gastric epithelium in general. In the present study, mRNA expression levels of *Atp4a*, part of the proton pump, showed a trend towards being higher in *H. suis* positive pigs, which may be somewhat surprising, since other studies have shown that *H. pylori* infection can inhibit acid secretion through down-regulation of the expression of H^+^/K^+^ ATPase, resulting in hypochlorhydria [14, 46, 47]. However, yet another group of studies have described that *H. pylori* infection can in fact also cause hyperchlorhydria [48, 49], depending on the distribution of bacteria within the stomach, the infection stage, the profile of cytokines produced by the local epithelial cells or immune cells, and the pattern of gastritis [31, 50]. Therefore, the effect of *H. suis* infection on the dynamic changes of expression of H^+^/K^+^ ATPase as well as the function of parietal cells in the pig stomach needs to be further explored in future experimental studies.

Interestingly, significantly elevated expression levels of *Shh* were demonstrated in *H. suis* positive animals compared to animals free of *H. suis*, suggesting that *H. suis* infection affects the *Shh* signalling pathway. Sonic, India, and Desert hedgehog are important members of the Hedgehog family, playing an essential role during regulation of differentiation and growth of many tissues and cells [51]. In the stomach of mammals, and especially in the stomachs of mice and humans, Shh has been described to serve as an important regulator in the differentiation of gastric epithelium and immune cells as well as gastric gland morphogenesis [17, 52]. An exclusive expression of Shh is detected in the parietal cells located at the gland-pit boundary in the human stomach, which has been proven to be co-localized with ATPase [17, 53]. *H. pylori* infection has been described to induce an overexpression of Shh in mice during the early stage of infection and Shh may have a progressive role in the development of gastric cancer [54–56]. In addition, other studies have provided evidence that gastrin and gastric acid can stimulate the expression of *Shh*, while *Shh* in turn is also important for maintaining acid secretion, suggesting a feedback mechanism between gastric acid and *Shh* expression [57, 58]. It is also worth noting that Shh signalling is crucial for macrophage infiltration in the stomach [55]. Indeed, higher numbers of macrophages have been detected in the fundic region of the stomach from BALB/c mice during the initial stages of *H. suis* infection [16].

In summary, an effective method for the isolation and culture of porcine parietal cells was established. Direct interactions between *H. suis* and parietal cells were investigated using this in vitro cell model as well as in vivo in the stomach of pigs at slaughter age. *H. suis* was shown to interfere with parietal cells, by directly affecting their viability in vitro. *H. suis* infection triggers abnormal mRNA expression levels of *Atp4a*, responsible for acid production and regulation. In addition, *H. suis* infection was shown to induce a marked up-regulation of transcriptional levels of *Shh*, a critical factor involved in gastric organogenesis, glandular differentiation, and gastric homeostasis.
